# High CTHRC1 expression may be closely associated with angiogenesis and indicates poor prognosis in lung adenocarcinoma patients

**DOI:** 10.1186/s12935-019-1041-5

**Published:** 2019-11-29

**Authors:** Yangshan Chen, Yu Sun, Yongmei Cui, Yiyan Lei, Neng Jiang, Wenting Jiang, Han Wang, Lili Chen, Jiping Luo, Yanyang Chen, Kejing Tang, Chengzhi Zhou, Zunfu Ke

**Affiliations:** 1 Department of Pathology, The First Affiliated Hospital, Sun Yat-sen University, No. 58, Zhongshan Second Road, Guangzhou, 510080 Guangdong People’s Republic of China; 2 Department of Thoracic Surgery, The First Affiliated Hospital, Sun Yat-sen University, Guangzhou, 510080 Guangdong People’s Republic of China; 3 Department of Respiratory Medicine, The First Affiliated Hospital, Sun Yat-sen University, Guangzhou, 510080 Guangdong People’s Republic of China; 4 State Key Laboratory of Respiratory Disease, National Clinical Research Center for Respiratory Disease, Guangzhou Institute of Respiratory Health, The First Affiliated Hospital of Guangzhou Medical University, Guangzhou, 510080 Guangdong People’s Republic of China; 5 Institute of Precision Medicine, The First Affiliated Hospital, Sun Yat-sen University, Guangzhou, 510080 Guangdong People’s Republic of China

**Keywords:** Lung adenocarcinoma (LUAD), Collagen triple helix repeat containing 1 (CTHRC1), Vascular endothelial growth factor (VEGF), Microvessel density (MVD), Tumor angiogenesis

## Abstract

**Background:**

This study aimed to investigate the prognostic value of the potential biomarker collagen triple helix repeat containing 1 (CTHRC1) in lung adenocarcinoma (LUAD) patients.

**Methods:**

A total of 210 LUAD patients diagnosed between 2003 and 2016 in the Department of Pathology of the First Affiliated Hospital of Sun Yat-sen University were included in this study. The expression of CTHRC1 and vascular endothelial growth factor (VEGF), and microvessel density (MVD, determined by CD34 immunostaining) were evaluated by immunohistochemistry in LUAD tissues. The association between the expression of these proteins and clinicopathological features or clinical outcomes was analyzed.

**Results:**

Here, we confirmed that CTHRC1 expression was associated with prognosis and can serve as a significant predictor for overall survival (OS) and progression-free survival (PFS) in LUAD. Additionally, we observed that CTHRC1 expression was positively associated with tumor angiogenesis markers, such as VEGF expression (*P *< 0.001) and MVD (*P *< 0.01). Then, we performed gene set enrichment analysis (GESA) and cell experiments to confirm that enhanced CTHRC1 expression can promote VEGF levels. Based on and cox regression analysis, a predictive model that included CTHRC1, VEGF and MVD was constructed and confirmed as a more accurate independent predictor for OS (*P *= 0.001) and PFS (*P *< 0.001) in LUAD than other parameters.

**Conclusions:**

These results demonstrated that high CTHRC1 expression may be closely related to tumor angiogenesis and poor prognosis in LUAD. The predictive model based on the CTHRC1 level and tumor angiogenesis markers can be used to predict LUAD patient prognosis more accurately.

## Background

According to the most recently reported global cancer statistics, lung cancer remains the leading cause of cancer-related deaths [1–3] and the mortality rate is 18.4% [1]. Lung adenocarcinoma (LUAD), the most commonly diagnosed histologic subtype of lung cancer [4], causes more than 500,000 deaths globally each year [5, 6]. Currently, the adenocarcinoma subtype of NSCLC had more effective responses to recently developed targeted therapies such as pemetrexed, gefitinib, bevaciuzumab, and crizotinib than the non-adenocarcinoma subtype [7–9]. Despite improvements in chemotherapeutic interventions and surgical resection, the prognosis for patients with LUAD remains poor, with a dismal 5-year survival rate of 16% [3], which is due to the high occurrence of tumor recurrence and distant metastases [10, 11]. Accordingly, the discovery of effective prognostic and diagnostic biomarkers in early stage will have a significant impact on further improving treatment outcomes in LUAD.

Collagen triple helix repeat containing 1 (CTHRC1) is a chondrocyte-specific, secreted glycoprotein that was originally discovered in a rat model of balloon-injured arteries [12–17]. The overexpression of CTHRC1 is frequently detected in several solid tumors, such as melanoma, breast ductal carcinoma, gastric cancer, hepatocellular carcinoma and colorectal cancer [18–22]. According to our previous studies, CTHRC1 overexpression was significantly correlated with metastasis in patients with non-small cell lung cancer (NSCLC) [23]. However, more evidence still needed to determine the prognostic value of CTHRC1 in LUAD, which is the most common subtype of lung cancer.

Angiogenesis, the process in which capillaries sprout from pre-existing vessels, has long been regarded as the principal mechanism of tumor vascularization, throughout cancer occurrence, growth, migration, invasion and distant metastasis in lung cancer [24–26]. Notably, Pyagay et al. [17] found that CTHRC1 expression cannot be detected in normal arteries but is only transiently expressed in injured arteries. Additionally, it was reported that the overexpression of CTHRC1 can significantly promote tumor angiogenesis in pancreatic tumors and gastrointestinal stromal tumors [27, 28]. However, the relationship between CTHRC1 expression and tumor angiogenesis in LUAD remains unclear.

Therefore, in the current study, we aimed to examine the association of CTHRC1 expression and tumor angiogenesis and to provide more significant evidence for its application in LUAD prognosis.

## Materials and methods

### Patients

A total of 210 LUAD patients were included in this study; patients were diagnosed between 2003 and 2016 in the Department of Pathology of the First Affiliated Hospital of Sun Yat-sen University and followed up for between 6 and 125 months (median 46.0 months). The patients’ clinical and tumor pathological characteristics are shown in Table 1. Formalin-fixed and paraffin-embedded (FFPE) cancer surgical specimens were used to perform subsequent analyses. This study was approved by the institutional ethics committee of the First Affiliated Hospital of Sun Yat-sen University, and written informed consent was obtained from all patients.

**Table 1:** Correlation of CTHRC1 and clinicopathologic characteristics in patients with LUAD

Clinicopathologic characteristics	All cases (n = 210)	CTHRC1 expression		*P*-value
		Low (n = 137)	High (n = 73)	
Gender				
Male	114	80	34	0.111
Female	96	57	39	
Age (years)				
≤ 50	64	39	25	0.432
> 50	146	98	48	
Smoking				
Yes	43	33	10	0.105
No	167	104	63	
Tumor differentiation				
Well	10	7	3	0.172
Moderately	181	114	67	
Poorly	19	16	3	
Clinical stage				
I	130	102	28	*< 0.001*
II	25	11	14	
III	36	18	18	
IV	19	6	13	
T classification				
T1	76	53	23	*0.025*
T2	112	76	36	
T3	12	5	7	
T4	10	3	7	
N classification				
N0	146	107	39	*0.001*
N1	21	9	12	
N2	33	18	15	
N3	10	3	7	
M classification				
M0	195	132	63	*0.011*
M1	15	5	10	

Fisher’s exact tests; Chi-square tests. Statistical significance (*P *< 0.05) is shown in italic

*LUAD* lung adenocarcinoma, *CTHRC1* collagen triple helix repeat containing 1, *TNM* tumor-node-metastasis

### Immunohistochemistry (IHC)

Representative paraffin-embedded tissues were arrayed with a tissue-arraying instrument with 2.0-mm diameter core and were sectioned (4 um) for further analysis. CTHRC1 (Abcam, Cambridge, UK) was used in a 1:100 dilution [29, 30], VEGF (ZSGB-Bio, Beijing, China) and CD34 (ZSGB-Bio, Beijing, China) was used in ready to use dilution [31]. Samples were incubated with antibodies against CTHRC1 (Abcam, Cambridge, UK), VEGF (ZSGB-Bio, Beijing, China) and CD34 (ZSGB-Bio, Beijing, China). The protocol for the IHC staining of tumor tissues from humans was described previously [32]. Brown particles in the cytoplasm represent CTHRC1- or VEGF- positive staining. The expression intensities of CTHRC1 and VEGF were semiquantitatively evaluated according to the immunostaining intensity and positive cell distribution. The percentage of positive tumor cells was determined in at least three areas at 400× magnification and was averaged. The mean percentage was then assigned to one of five categories (Additional file 1: Fig. S1a–j): 0, no cancer cells stained; 1, 0–10% of cancer cells stained; 2, 11–50% of cancer cells stained; 3, 51–75% of cancer cells stained, 4, more than 75% of cancer cells stained. The intensity of immunostaining was scored as follows: 0, colorless; 1, tan; 2, brownish-yellow; and 3, dark brown. A weighted score was obtained by multiplying the positive cell percentage and staining intensity for each case. Microvessel density (MVD) was evaluated by the technique of Weidner et al. [33] and was based on the average CD34 positive cell count from IHC staining. Tumor slides were scanned first at low magnification (100×) to select three fields with the highest vascularization where the cell membrane of vascular endothelial cells was present and (or) there was brown staining, and then the microvessels were counted at high magnification (400×) (Additional file 1: Fig. S1k–o). Microvessels with a clearly defined lumen or a well-defined linear vessel shape were selected for counting and branching vessel structures were regarded as a single vessel. The mean value of three fields was considered as the microvessel density (MVD) for each case. Based on the receiver operative characteristic (ROC) analysis, the optimal cutoff value of CTHRC1, VEGF and MVD was confirmed: a staining index of 7.5 and 5 or greater was used to define tumors with high CTHRC1 and VEGF expression, respectively, and a staining index below 7.5 or 5 was defined as low expression; an evaluation of 28.5 or greater was used to define tumors with a high MVD, while an evaluation below 28.5 was used to define tumors with a low MVD [23, 34, 35].

### Gene set enrichment analysis (GSEA)

JavaGSEA-3.0 was downloaded from the official website. Before performing the analysis, we downloaded the related Molecular Signatures Database (MSigDB) and obtained microarray data from 40 LUAD patients and from the microarray data of 514 LUAD cases from The Cancer Genome Atlas (TCGA) database (https://www.cbioportal.org/, accessed September 18, 2019). These two cohorts were divided into a high-CTHRC1 group and a low-CTHRC1 group based on the level of CTHRC1 expression. GSEA was then carried out to determine the functions or pathways that showed statistically significant, concordant differences between the two groups. A positive enrichment score (ES) and a normalized enrichment score (NES) indicate that the majority of genes in this gene set were positively associated with our predefined group statuses. A normalized *P*-value (NOM *P*-value) of < 0.05 was considered statistically significant.

### Cell lines

Primary normal lung epithelial cells (BEAS-2B) were purchased from American Type Culture Collection (ATCC) and cultured in a keratinocyte serum-free medium (Invitrogen, Carlsbad, CA) supplemented with epidermal growth factor (EGF) (Invitrogen), bovine pituitary extract, and antibiotics (100 μg/mL streptomycin and 100 U/mL penicillin). Lung adenocarcinoma cell lines (A549, GLC-82, SPC-A1, PC9, H1299 and H1975) and non-adenocarcinoma cell lines (L78 and H460) were maintained in Dulbecco’s modified Eagle’s medium (DMEM; Invitrogen, USA) supplemented with 10% fetal bovine serum (HyClone, San Angelo, TX, USA).

### Stable clone establishment

CTHRC1 shRNA vectors (GV248, CTHRC1-shRNA, and Control shRNA) and CTHRC1-overexpression vectors (GV358, CTHRC1, and Control vector) were purchased from GeneChem (Shanghai, China), and were manipulated according to the protocol provide by the manufacturer. The transfection efficiency of the template was detected by fluorescence microscope (Axio Observer Z1, Zeiss), western blotting and quantitative real-time PCR.

### Western blotting (WB)

Western blotting was performed as previously described [32]. Targeted membranes were incubated with the antibodies for CTHRC1 (Abcam, USA), VEGFA (Abcam, USA) and Actin (Cell Signaling Technology, USA) in 5% milk/tris-buffered saline Tween-20 (TBST) at 4 °C overnight, and then were washed with TBST and incubated with horseradish peroxidase (HRP)-conjugated secondary antibodies (Cell Signaling Technology, USA) for 1 h with shaking. After enhanced chemiluminescence (ECL) (Merck Millipore, Germany) reaction, the immunoreactive bands were observed through a Gel Imaging System (Syngene, USA).

### Total RNA extraction and Quantitative Real-time PCR (qRT-PCR)

Total RNA from tissue specimens and cells was extracted with TRIzol method Invitrogen, USA). RNA was reverse transcribed into cDNA using the two-step method with PrimeScript™ RT reagent kit with gDNA Eraser (Takara, China), according to the manufacturer’ s instructions. Then, qRT-PCR was performed with the SYBR^®^ Premix Ex TaqTM kit (Takara, China), according to the manufacturer’ s protocol. The following primers were used: CTHRC1, forward 5′-TGGACACCCAACTACAAGCA-3′ and reverse 5′-GAACAAGTGCCAACCCAGAT-3′; GAPDH, forward 5′-ACCCACTCCTCCACCTTTG-3′ and reverse 5′-CTCTTGTGCTCTTGCTGGG-3′. GAPDH was used as an internal reference, with the 2^−ΔΔCt^ method used for quantitation [36].

### Statistical analysis

Statistical analysis was performed with SPSS 16.0 (SPSS Inc., Chicago, IL, USA). A two-tailed *p*-value of < 0.05 was considered statistically significant. Bonferroni correction was used to adjust the statistical significance level in multiple testing. Fisher’s exact tests, Chi-square tests, a Student’s t-test and one-way ANOVA were used for comparisons between groups. The MVD was presented as the mean ± the standard deviation (SD). The concordance rate of linear regression plots was assessed using the Pearson test. Receiver operative characteristic (ROC) analysis was performed to select the optimal cutoff value and to determine the predictive value of the factors. The Kaplan–Meier method was used to examine the overall survival (OS) and progression-free survival (PFS), and the log-rank test was used to compare curves for two or more groups. To investigate the independent prognostic factors for OS and PFS, a Cox proportional hazard regression model was applied.

## Results

### Clinicopathologic patient characteristics

The clinical and histopathologic characteristics of the 210 LUAD patients are summarized in Table 1. The patient cohort consisted of 114 (54.3%) males and 96 (45.7%) females, and the mean age at diagnosis was 58.5 years (range, 33–81 years). Of the 210 patients with a known smoking status, most (79.5%) were nonsmokers. The tumor size was 2.67 ± 1.8 cm (range, 0.4–15 cm). Tumors were classified as well, moderately and poorly differentiated for 10 (4.8%), 181 (86.2%) and 19 (9.0%) patients, respectively. The number of patients with pathologic TNM stage I, II, III, and IV was 130 (61.9%), 25 (11.9%), 36 (17.1%) and 19 (9.0%), respectively.

### Association between CTHRC1 expression and clinicopathologic characteristics in LUAD

**Fig. 1:**
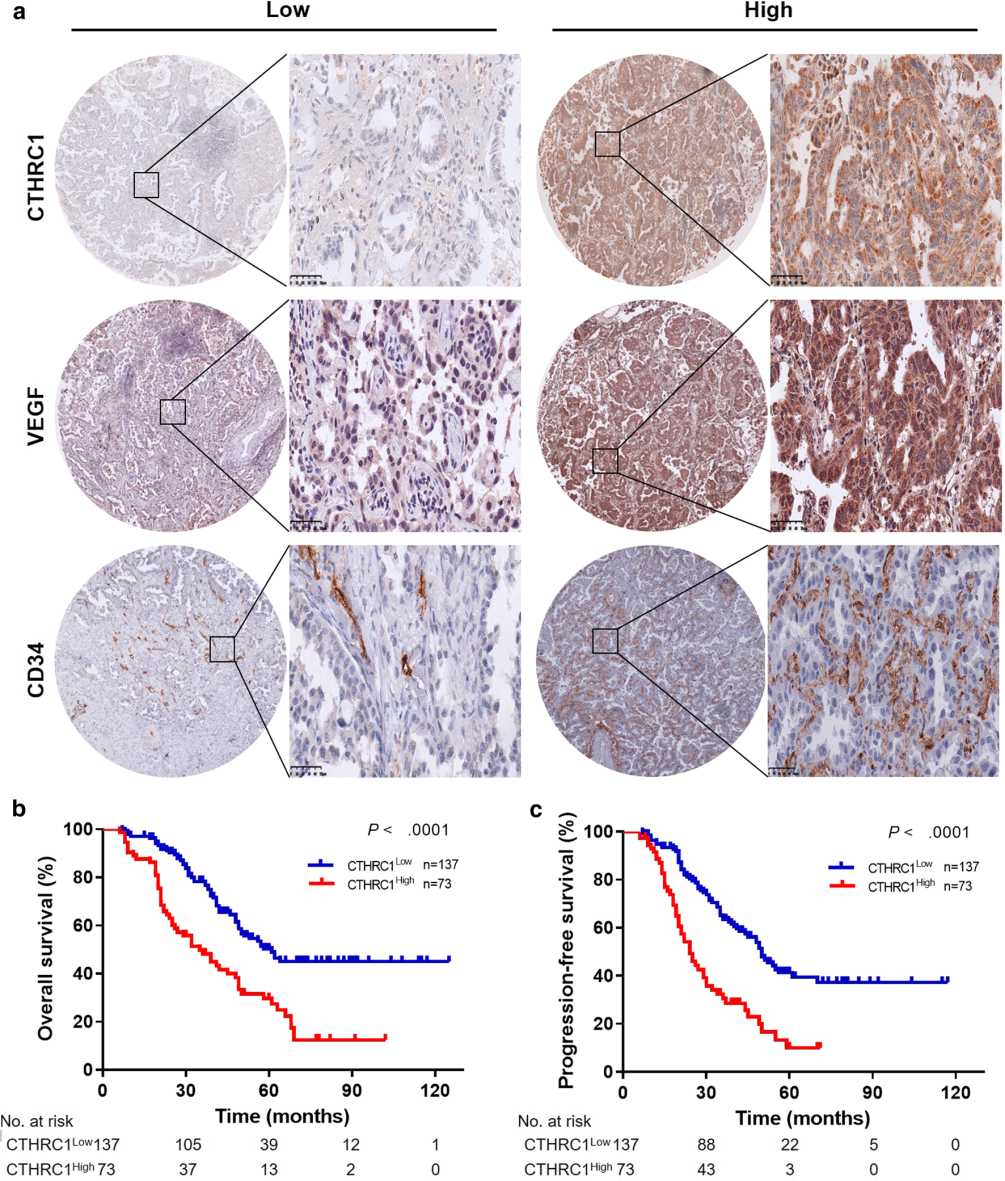
CTHRC1 expression in LUAD and its correlation with patient survival. **a** Representative immunochemistry (IHC) staining for CTHRC1, VEGF and CD34 in an LUAD tissue microarray (2.0-mm cores). Scale bar: 50 µm. Kaplan–Meier survival curves for **b** OS and **c** PFS according to CTHRC1 expression (*P *< 0.001). CTHRC1, collagen triple helix repeat containing 1; VEGF, vascular endothelial growth factor; MVD, microvessel density; OS, overall survival; PFS, progression-free survival; LUAD, lung adenocarcinoma

Representative stained fields from histopathological slides for CTHRC1, VEGF and CD34 are displayed in Fig. 1a and Additional file 1: Fig. S1. As shown in Table 1, the CTHRC1 expression status in LUAD was significantly associated with the clinical stage (*P *< 0.001), T classification (*P *< 0.001), N classification (*P *< 0.001) and M classification (*P *< 0.001). In contrast, there were no significant correlations between CTHRC1 expression and other clinicopathologic characteristics.

### Association between CTHRC1 expression and patient survival in LUAD

Univariate analysis of clinicopathologic features demonstrated that age, clinical stage, T classification, N classification, M classification and CTHRC1 expression in LUAD were significantly correlated with overall survival (OS) (*P *< 0.05; Table 2), while clinical stage, N classification, M classification and CTHRC1 expression in LUAD were significantly correlated with progression-free survival (PFS) (*P *< 0.05; Table 2). According to the Kaplan–Meier analysis, the OS and PFS of patients in the CTHRC1^High^ group were significantly shorter than those in the CTHRC1^Low^ group (*P *< 0.001; Fig. 1b, c). Then, we performed a Kaplan–Meier survival analysis according to clinical stage, T classification, N classification and M classification and the data showed that clinical stage, N classification and M classification were significantly related to patient survival, and the survival time in the CTHRC1^High^ group was distinctly shorter than that in the CTHRC1^Low^ group (*P *< 0.01; Additional file 1: Fig. S2, Additional file 2: Table S1–S3).

**Table 2:** Univariate analysis of factors associated with overall survival and progression-free survival in LUAD patients

Variables	OS			PFS		
	Hazard ratio	95% CI	*P*-value	Hazard ratio	95% CI	*P*-value
Gender	1.067	0.742–1.534	0.727	1.090	0.759–1.566	0.640
Age	1.050	1.032–1.069	*<* *0.001*	1.504	0.993–2.278	0.054
Smoking	0.990	0.637–1.541	0.966	0.981	0.631–1.525	0.931
Tumor differentiation	0.724	0.450–1.166	0.184	0.897	0.574–1.400	0.631
Clinical stage	2.572	2.112–3.132	*<* *0.001*	2.481	2.034–3.025	*<* *0.001*
T classification	1.414	1.106–1.808	*0.006*	1.163	0.900–1.504	0.248
N classification	1.935	1.608–2.330	*<* *0.001*	1.793	1.480–2.172	*<* *0.001*
M classification	7.165	3.673–13.976	*<* *0.001*	6.873	3.432–13.766	*<* *0.001*
CTHRC1 expression	2.300	1.600–3.308	*<* *0.001*	2.643	1.827–3.822	*<* *0.001*
VEGF expression	2.733	1.860–4.017	*<* *0.001*	3.673	2.485–5.429	*<* *0.001*
MVD count	2.122	1.476–3.050	*<* *0.001*	1.881	1.305–2.711	*0.001*

*CI* confidence interval; statistical significance (*P *< 0.05) is shown in italic

*LUAD* lung adenocarcinoma, *OS* overall survival, *PFS* progression-free survival, *CTHRC1* collagen triple helix repeat containing 1, *VEGF* vascular endothelial growth factor; *MVD* microvessel density, *TNM* tumor-node-metastasis

**Fig. 2:**
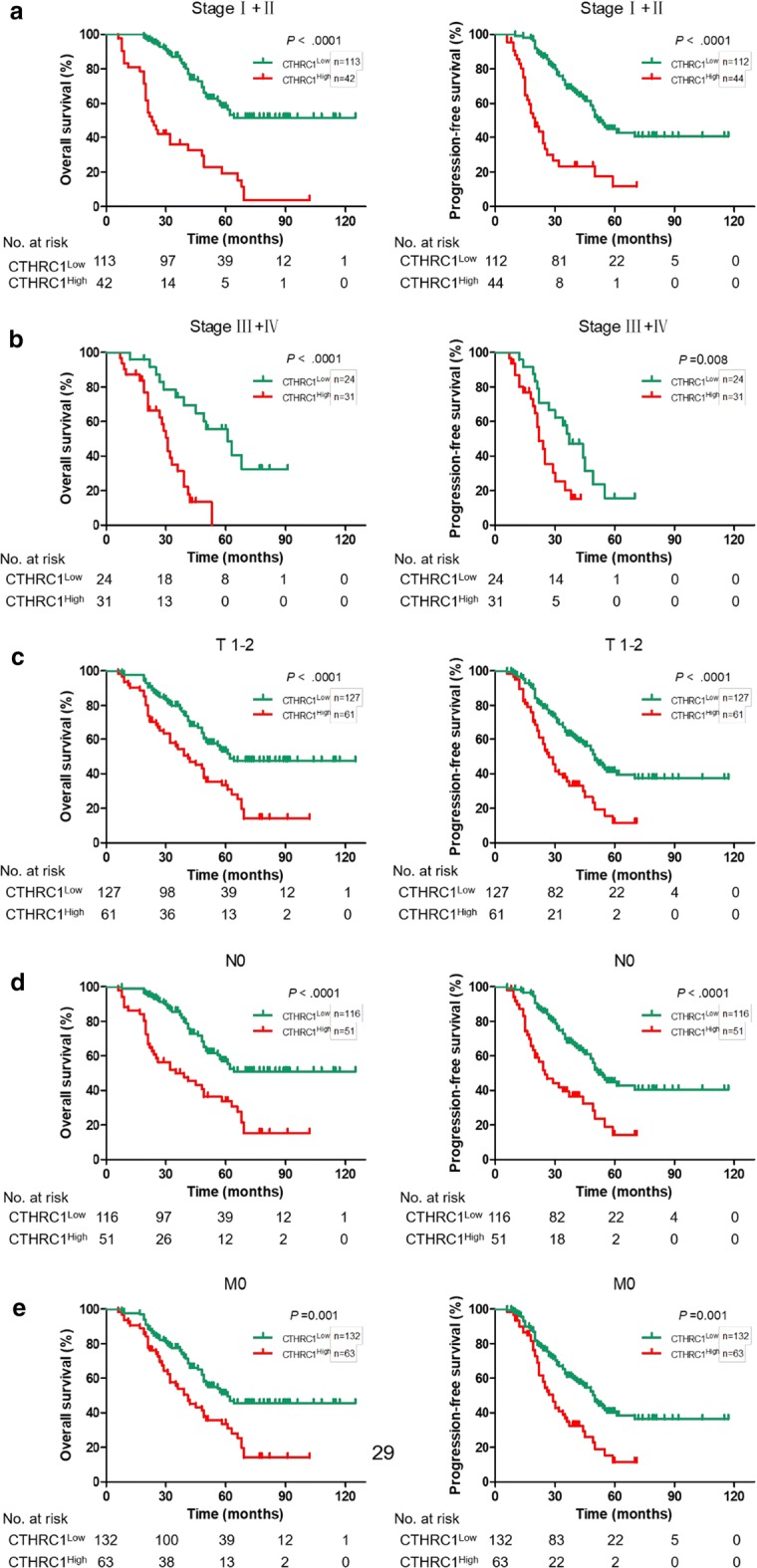
Survival curves stratified by the CTHRC1 status according to clinical stage and T, N, and M classifications. In the **a** stage I + II subgroup, **b** stage III + IV subgroup, **c** T1–2 subgroup, **d** N0 subgroup and **e** M0 subgroup, patients in the CTHRC1-low group showed a significantly better OS and PFS than patients in the CTHRC1-high group. CTHRC1, collagen triple helix repeat containing 1; OS, overall survival; PFS, progression-free survival; LUAD, lung adenocarcinoma

As shown in Table 3, multivariate Cox regression analysis revealed that CTHRC1 expression was an independent prognostic marker for LUAD. The prognostic value of CTHRC1 expression was investigated according to clinical stage, T classification, N classification and M classification. Our results showed that the expression of CTHRC1 was strongly associated with the OS and PFS of patients in the clinical stage s I + II, clinical stages III + IV, T1–2, N0 and M0 subgroups (*P *< 0.05; Fig. 2). However, no significant difference was observed between the CTHRC1^High^ group and the CTHRC1^Low^ group in the T3–4, N1–3 and M1 subgroups, probably because there was a limited number of samples in these subgroups (Additional file 1: Fig. S3). We concluded that patients with tumors expressing high CTHRC1 levels had distinctly poor survival compared with patients with low CTHRC1 levels in the early or advanced stage subgroups (*P *< 0.05; Fig. 2a, b). Similarly, there was clearly shorter OS and PFS in patients with high CTHRC1 expression in the T1–2 subgroup (*P *< 0.05; Fig. 2c), N0 subgroup (*P *< 0.05; Fig. 2d) and M0 subgroup (*P *< 0.05; Fig. 2e). Therefore, CTHRC1 may act as an effective prognostic marker for LUAD patients.

**Table 3:** Multivariate analysis of factors associated with overall survival and progression-free survival in LUAD patients

Variables	OS			PFS		
	Hazard ratio	95% CI	*P*-value	Hazard ratio	95% CI	*P*-value
Gender	1.074	0.703–1.643	0.740	1.204	0.790–1.834	0.389
Age	3.223	1.917–5.418	*<* *0.001*	2.097	1.344–3.273	*0.001*
Smoking	0.934	0.563–1.588	0.833	0.892	0.548–1.451	0.644
Tumor differentiation	0.946	0.563–1.588	0.833	1.318	0.802–2.164	0.276
Clinical stage	2.996	2.135–4.204	*<* *0.001*	3.267	2.302–4.638	*<* *0.001*
T classification	0.805	0.673–1.016	0.067	0.730	0.576–0.925	*0.009*
N classification	0.911	0.665–1.250	0.565	0.796	0.576–1.100	0.167
M classification	1.433	0.628–3.269	0.393	1.163	0.504–2.686	0.732
CTHRC1 expression	1.097	1.029–1.170	*0.004*	1.133	1.061–1.211	*<* *0.001*
VEGF expression	0.987	0.927–1.051	0.680	1.033	0.970–1.100	0.313
MVD count	1.015	0.999–1.030	0.060	1.005	0.990–1.020	0.507

*CI* confidence interval; statistical significance (*P *< 0.05) is shown in italic

*LUAD* lung adenocarcinoma, *OS* overall survival, *PFS* progression-free survival, *CTHRC1* collagen triple helix repeat containing 1, *VEGF* vascular endothelial growth factor, *MVD* microvessel density, *TNM* tumor-node-metastasis, *CI* confidence interval

### Association between CTHRC1 expression and tumor angiogenesis markers

**Fig. 3:**
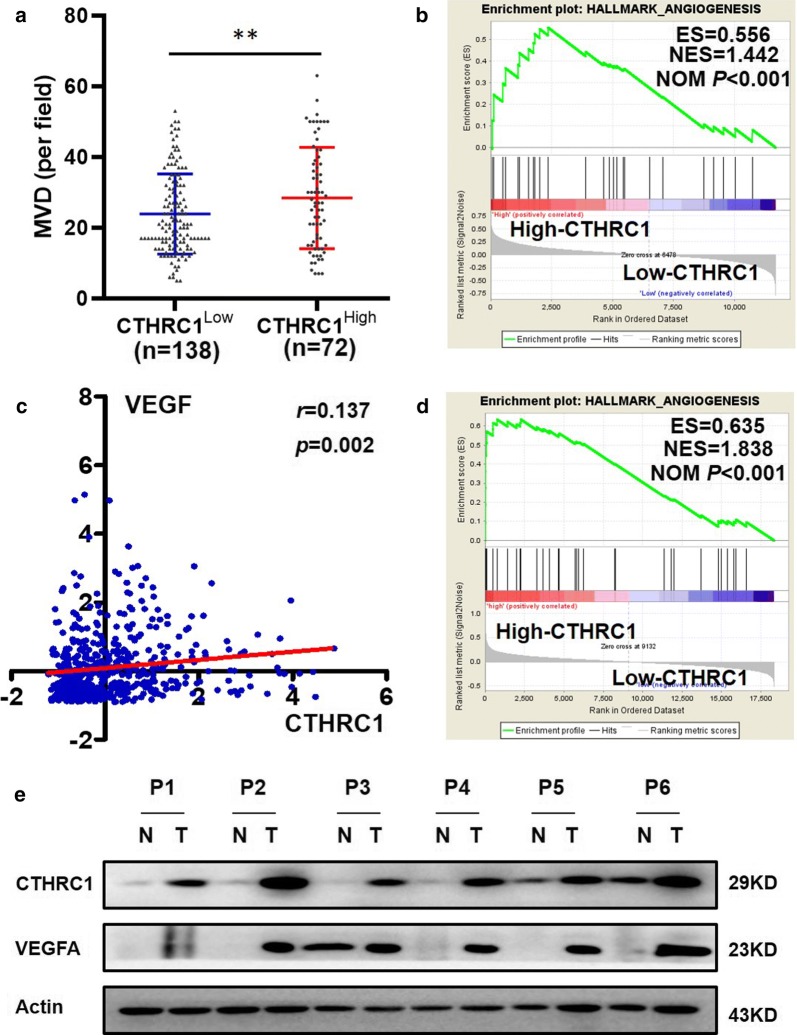
CTHRC1 expression was potentially associated with tumor angiogenesis markers in LUAD. **a** Patients with high CTHRC1 expression had a higher MVD count (mean: 23.9 vs. 28.4; **P < 0.01). **b** GSEA for angiogenesis between the high-CTHRC1 group and the low-CTHRC1 group in LUAD. **c** Scatter plot showing the correlation log fold change between CTHRC1 and VEGF in TCGA patients with LUAD (n = 514; *r *= 0.537, *P *= 0.002). **d** GSEA results of angiogenesis gene sets for the high-CTHRC1 expression group in TCGA patients with LUAD. **e** WB analysis of CTHRC1 and VEGFA expression in each of the primary LUAD tissue (T) and corresponding non-cancerous tissue (N). GSEA, Gene Set Enrichment Analysis; ES, enrichment score; NES, normalized enrichment score; NOM P-value, normalized P-value; TCGA, the Cancer Genome Atlas; WB, western blotting; CTHRC1, Collagen triple helix repeat containing 1; VEGFA, vascular endothelial growth factor A; LUAD, lung adenocarcinoma

We found that patients in the CTHRC1^High^ group expressed more VEGF (*P *< 0.001; Table 4) and had a higher MVD (*P *= 0.001; Fig. 3a, Table 4) than those in the CTHRC1^Low^ group. To elucidate the relationship between CTHRC1 expression and tumor angiogenesis, we utilized Gene Set Enrichment Analysis (GSEA) to determine whether a previously defined set of angiogenesis-associated genes showed statistically significant, concordant differences between the high-CTHRC1 group and the low-CTHRC1 group (ES = 0.556, NES = 1.442; *P *< 0.001; Fig. 3b) in our microarray data. The results showed that angiogenesis-associated genes were enriched in the high-CTHRC1 group, which indicated that the expression of genes related to the promotion of angiogenesis was more activated in LUAD patients with high CTHRC1 levels than in LUAD patients with low CTHRC1 levels. Additionally, in the TCGA cohort, we observed that the mRNA expression of CTHRC1 was positively associated with the expression of VEGF (*r *= 0.137, *P *= 0.002; Fig. 3c), and GSEA also showed that CTHRC1 expression remained linked with angiogenesis (ES = 0.635, NES = 1.838; *P *< 0.001; Fig. 3d). As shown in Fig. 3e, CTHRC1 and VEGFA was also differentially increased in six detected LUAD samples compared to corresponding non-cancerous tissues. These results further demonstrated that high CTHRC1 expression may be closely associated with tumor angiogenesis due to the upregulation of angiogenesis-related genes.

**Table 4:** Correlation of CTHRC1 expression with VEGF expression and MVD

Characteristics	CTHRC1 expression		*P*-value
	Low (n = 137)	High (n = 73)	
VEGF expression			
Low	85 (80.2%)	21 (19.8%)	< 0.001^a^
High	52 (50.0%)	52 (50.0%)	
MVD counts			
Low	96 (71.6%)	38 (28.4%)	0.011^a^
High	41 (53.9%)	35 (46.1%)	

*CTHRC1* collagen triple helix repeat containing 1, *VEGF* vascular endothelial growth factor, *MVD* microvessel density

^a^Chi-square test, *P *< 0.05

### Enhanced CTHRC1 expression can promote VEGF expression in LUAD in vitro

**Fig. 4:**
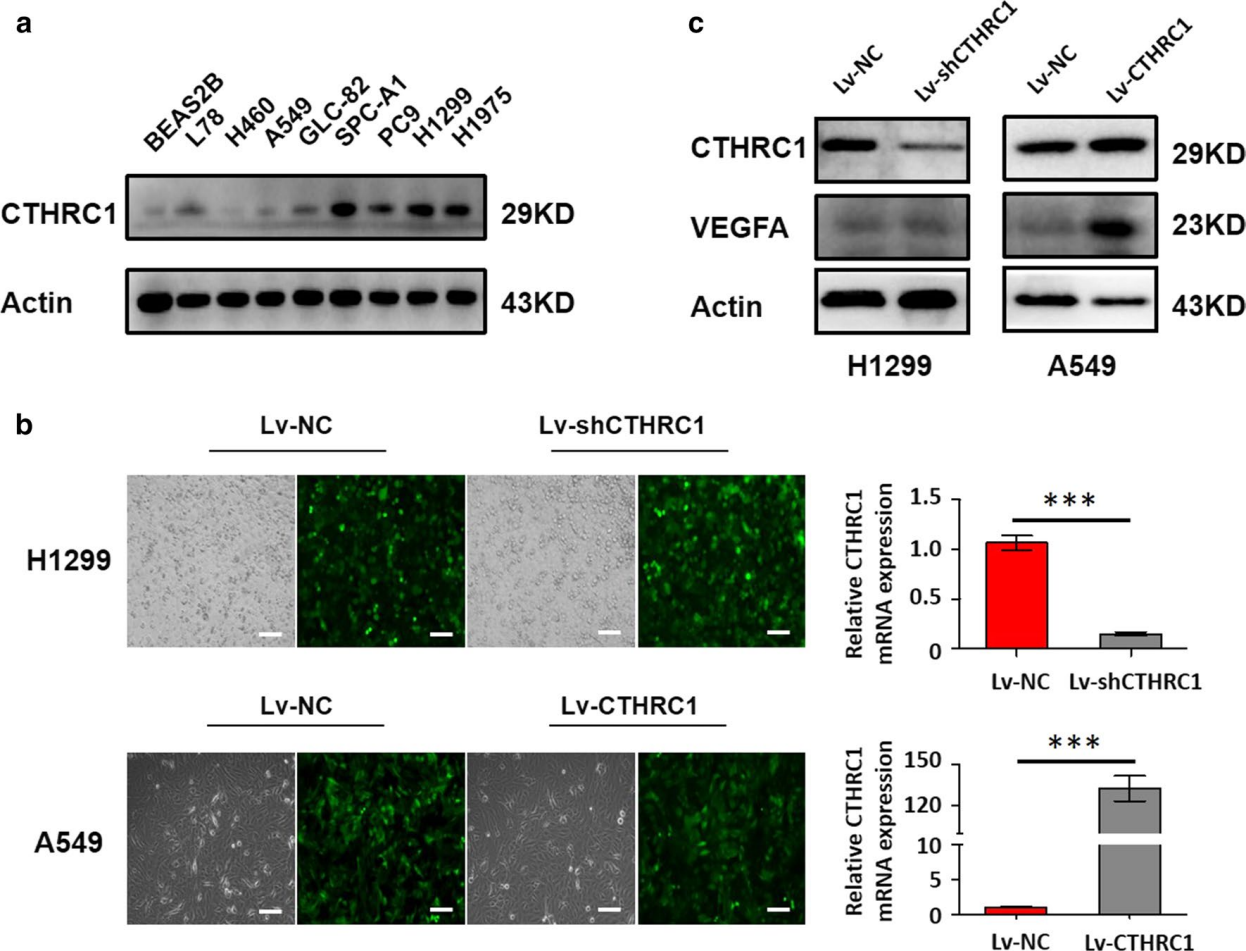
Enhanced CTHRC1 expression can promote VEGF expression in LUAD in vitro. **a** CTHRC1 protein was detected by WB in BEAS-2B, lung non-adenocarcinoma cell lines (L78 and H460) and lung adenocarcinoma cell lines (A549, GLC-82, SPC-A1, PC9, H1299 and H1975). **b** GFP expression after H1299 and A549 transfection with the high-expressions CTHRC1 lentivirus and the shCTHRC1 lentivirus respectively. CTHRC1 mRNA expression was analyzed by using the qRT-PCR. Scale bar: 100um. Error bars represent mean ± SD from three independent experiments (***P < 0.001). **c** CTHRC1 and VEGFA protein expression was analyzed by using the WB in the constructed Lv-shCTHRC1 H1299 cells and Lv-CTHRC1 A549 cells. GFP, Green fluorescent protein; qRT-PCR, quantitative real-time PCR; SD, standard deviation; CTHRC1, Collagen triple helix repeat containing 1; VEGFA, vascular endothelial growth factor A; LUAD, lung adenocarcinoma

As shown in Fig. 4a, lung adenocarcinoma cell lines, including A549, GLC-82, SPC-A1, PC9, H1299 and H1975, exhibited higher CTHRC1 levels compared to that of primary human normal lung epithelial cells (BEAS-2B) and non-adenocarcinoma cell lines (L78 and H460). To investigate the angiogenesis marker expression on CTHRC1 enhancement or deregulation LUAD cells, we constructed stable clone of H1299-shCTHRC1 cells and A549-CTHRC1 cells by using lentivirus transfection. At 72 h after transfection with Lv-shCTHRC1 or Lv-CTHRC1 and their corresponding control virus, respectively, H1299 and A549 were observed by fluorescence microscopy. Green fluorescent protein (GFP) was expressed by more than 80% of the cells (Fig. 4b). After stable transfection and puromycin screening, CTHRC1 mRNA expression and protein levels in the H1299-Lv-shCTHRC1 group were significantly lower than that in the control group (0.147 ± 0.015 versus 1.066 ± 0.061, *P *< 0.001; Fig. 4b, c), while CTHRC1 mRNA expression and protein levels in the A549-Lv-CTHRC1 group were significantly higher than that in the control group (132.6 ± 7.540 versus 1.035 ± 0.039, *P *< 0.001; Fig. 4b, c). Furthermore, the protein levels of VEGFA also presented significantly higher in the A549-Lv-CTHRC1 group than in the control group (Fig. 4c). These data demonstrated that enhanced CTHRC1 expression can promote VEGF expression in LUAD in vitro.

### Combining CTHRC1 expression with tumor angiogenesis markers to construct a predictive model

Univariate analysis of clinicopathologic characteristics demonstrated that tumor angiogenesis markers (VEGF and MVD) were significantly associated with OS (*P *< 0.05; Table 2) and PFS (*P *< 0.05; Table 2). Based on our results described above, it is reasonable to simultaneously consider CTHRC1 expression and tumor angiogenesis markers as a predicted panel. Therefore, we established a predictive model that included CTHRC1, VEGF and MVD, and all patients were divided into three groups: patients were assigned to the low-risk group if the three biomarkers were low; to the moderate-risk group if more than one of the three biomarkers was high; and to the high-risk group if the three biomarkers were high.

### CTHRC1 expression concomitant with VEGF expression and MVD predicts prognosis in LUAD

**Fig. 5:**
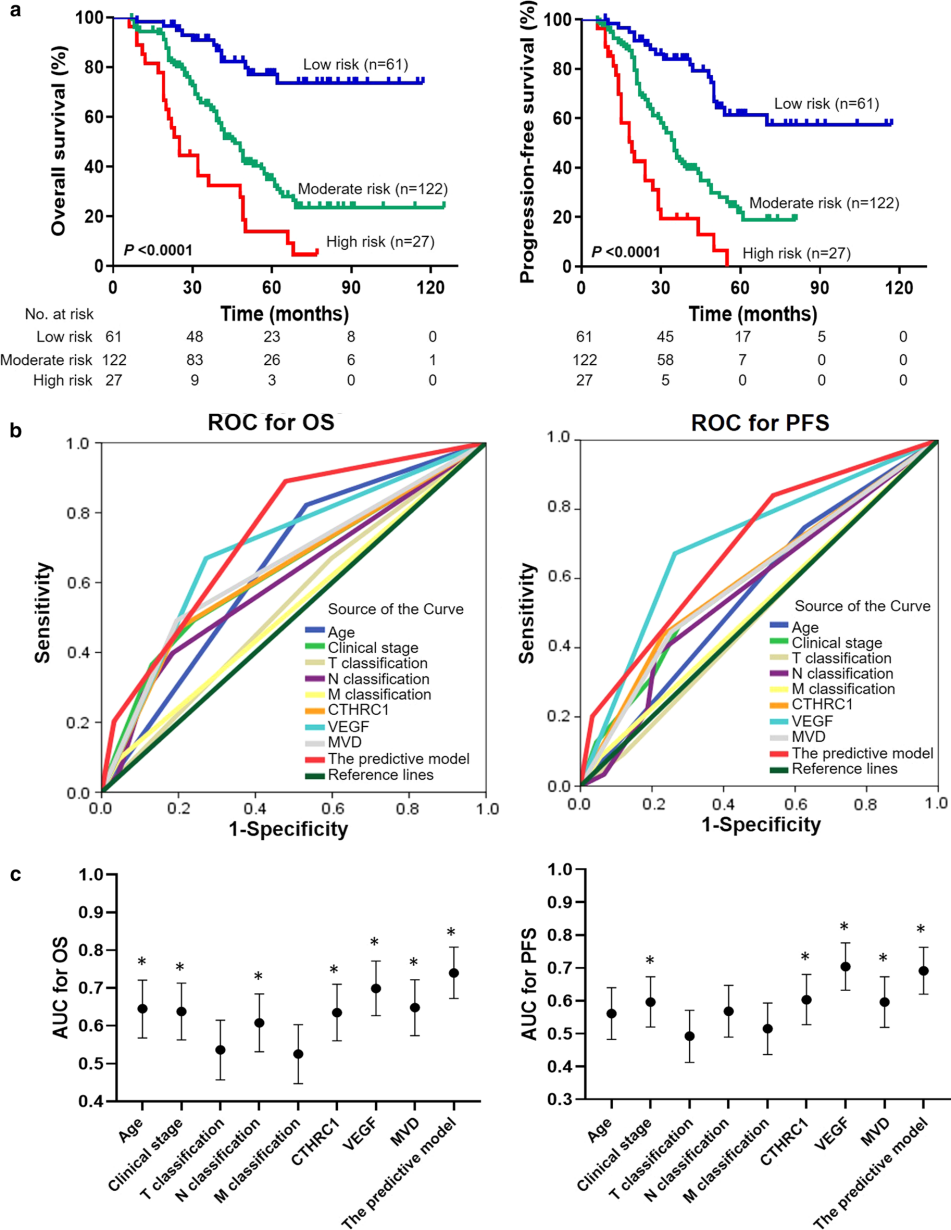
Validation of the predictive model. **a** Kaplan–Meier analysis of OS and PFS for the predictive model in 210 LUAD patients. **b** The predictive ability of the predictive model compared with single markers and other clinical prognostic parameters by ROC for OS and PFS. **c** The AUC with the 95% CI for OS and PFS (*P < 0.05). The details for the AUC and 95% CI are also shown in Additional file 2: Table S5. CTHRC1, collagen triple helix repeat containing 1; VEGF, vascular endothelial growth factor; MVD, microvessel density; OS, overall survival; PFS, progression-free survival; LUAD, lung adenocarcinoma; ROC, receiver operating characteristic; AUC, area under the curve

According to the Kaplan–Meier survival analysis, the three survival curves were significantly different, and high-risk patients had a shorter OS and PFS than those in the low-risk group and moderate-risk group (*P *< 0.001; Fig. 5a, Additional file 2: Table S4). Similarly, in multivariable analysis, the predictive model was also confirmed as an independent predictor of OS and PFS (*P *< 0.05; Table 5). The predictive power for OS of the predictive model was better, with an area under the curve (AUC) of 0.740 (95% confidence interval [CI] 0.672–0.808; *P *< 0.001; Fig. 5b, c, Additional file 2: Table S5), than other single biomarkers, such as age, clinical stage, T classification, N classification, M classification, CTHRC1, VEGF and MVD, as shown by the ROC analysis. For PFS, although the predictive power of VEGF was the best, with an AUC of 0.704 (95% CI 0.632–0.776; *P *< 0.01; Fig. 5b, c, Additional file 2: Table S5), VEGF was not an independent predictor of prognosis (*P *> 0.05; Table 3). Therefore, as an effective predictor for PFS (*P *< 0.001; Table 5) in LUAD, the predictive model had the best predictive value, with an AUC of 0.691 (95% CI 0.620–0.763; *P *< 0.001; Fig. 5b, c, Additional file 2: Table S5).

**Table 5:** Multivariate analysis of the predictive model with clinicopathologic characteristics in LUAD patients

Variables	OS			PFS		
	Hazard ratio	95% CI	*P*-value	Hazard ratio	95% CI	*P*-value
Gender	0.880	0.571–1.357	0.564	1.088	0.713–1.662	0.695
Age	1.062	1.042–1.084	*<* *0.001*	1.029	1.010–1.049	*0.003*
Smoking	1.071	0.655–1.752	0.784	0.878	0.541–1.427	0.601
Tumor differentiation	0.942	0.557–1.593	0.823	1.411	0.869–2.289	0.164
Clinical stage	2.792	1.990–3.918	*<* *0.001*	3.165	2.236–4.480	*<* *0.001*
T classification	0.840	0.664–1.063	0.146	0.727	0.575–0.920	*0.008*
N Classification	0.935	0.678–1.288	0.680	0.766	0.554–1.059	0.107
M Classification	1.633	0.729–3.661	0.233	1.318	0.869–2.289	0.517
The predictive model	1.795	1.291–2.496	*0.001*	2.301	1.671–3.168	*<* *0.001*

*CI* confidence interval; statistical significance (*P *< 0.05) is shown in italic

*LUAD* lung adenocarcinoma, *OS* overall survival, *PFS* progression-free survival, *TNM* tumor-node-metastasis, *CI* Confidence interval

## Discussion

Since CTHRC1 was first identified in a screen for differentially expressed genes in balloon-injured rats [17], an increasing number of scientists have investigated the prognostic value of CTHRC1 in tumors. Liu et al. [37] found that patients with higher CTHRC1 expression exhibited a remarkably shorter OS in four different pancreatic ductal adenocarcinoma (PDAC) cohorts. Additionally, some reports have demonstrated that patients with higher CTHRC1 levels tend to have poor prognosis in many tumors, such as Wilm’s tumor, esophageal squamous cell carcinoma, colorectal cancer and cervical squamous cell carcinoma [29, 38–40]. Similarly, we have previously analyzed the relationship between CTHRC1 expression and clinicopathologic features in NSCLC and found that higher CTHRC1 expression predicted poor prognosis [23]. However, large sample studies are scarce that focus on CTHRC1 expression and LUAD, which is the most common diagnostic subtype of lung cancer. In this study, we used a large number of clinical samples to verify the strong relationship between the CTHRC1 expression status and LUAD prognosis. Moreover, our results showed that higher CTHRC1 expression can serve as an independent predictive biomarker for poor OS and PFS in LUAD.

During tumorigenesis and development, tumor tissue is often under hypoxic and hyponutrition conditions [41]. There is also a volume of work demonstrating that under these conditions, a large number of new vessels will form due to the activation of angiogenic factors that are secreted by cancer cells, providing the oxygen and nutrients needed for tumor growth [41–43]. VEGF has been identified as the most important factor of many angiogenic factors related to tumor growth, indicating that VEGF could be a critical target for antiangiogenic therapy [44–46]. Wei et al. [47] indicated that neoadjuvant bevacizumab (a humanized anti-vascular endothelial growth factor (anti-VEGF) monoclonal antibody) in combination with chemotherapy appeared to be effective and safe in patients with unresectable stage III LUAD. However, few investigations have studied the clinical applications of anti-VEGF therapy in large LUAD samples, probably because of the difficulty in the early diagnosis of LUAD. Thus, an effective predictive panel is urgently needed to increase the accuracy of early diagnosis and to promote the development of anti-VEGF therapy in LUAD.

CTHRC1 can be upregulated to promote tumor growth (in vitro and in vivo) by several mechanisms, such as the demethylation of the CTHRC1 promoter and canonical WNT signaling, and can be inhibited by a group of microRNAs to reduce cancer growth (in vitro and in vivo) in many tumors, such as gastric cancer, colorectal cancer and oral cancer [14, 18, 48–50]. However, little is known about the regulation and function of CTHRC1 in the cancer microenvironment. Recently, CTHRC1 overexpression was reported to be associated with MVD and to induce the migration and tube formation of human umbilical vein endothelial cells (HUVECs) by increasing the phosphorylation of extracellular-signal-regulated protein kinase (ERK) and c-Jun N-terminal kinase (JNK) in gastrointestinal stromal tumors [28]. The administration of a CTHRC1-neutralizing inhibitor to a xenograft mouse model reduced the tumor burden and infiltration of Tie2-expressing monocytes (TEMs) in pancreatic tumor specimens, indicating that blocking the CTHRC1/angiopoietin-2 (Ang-2)/TEM axis during angiogenesis suppresses tumorigenesis [27]. Specifically, Zhang et al. [51] discovered that CTHRC1 activated hypoxia-inducible factor 1α (HIF-1α) and VEGF by regulating the phosphoinositide-3-kinase/protein kinase B/mammalian target of rapamycin (PI3K/AKT/mTOR) pathway, and the knockdown of CTHRC1 resulted in the repression of hepatitis B virus (HBV)-associated carcinogenesis in nude mice. However, the relationship between CTHRC1 expression and tumor angiogenesis and tumor microenvironment in LUAD is still unclear. This encourages our future work to further explore the underlying mechanisms of how high CTHRC1 expression promotes angiogenesis in LUAD.

In this study, we observed that CTHRC1 expression, an independent predictor of prognosis in LUAD, was positively associated with tumor angiogenesis markers, and the GSEA data provided more evidence that gene expression related to the promotion of angiogenesis was more activated in the high-CTHRC1 group with LUAD than in the low-CTHRC1 group. Furthermore, we validated that enhanced CTHRC1 expression can promote VEGF expression in LUAD in vitro. Based on the aforementioned results, it was reasonable to combine CTHRC1 and tumor angiogenesis markers to construct a predictive model in LUAD. Additionally, we found that the constructed predictive model can be used as a more accurate prognostic panel, as it exhibited the best predictive power for OS and PFS in LUAD patients. However, the specific functional mechanism in this study remains to be studied, and we will further explore the potential mechanism between CTHRC1 expression and tumor angiogenesis in LUAD in our next study.

## Conclusions

In summary, high CTHRC1 expression may be closely associated with angiogenesis, and the combination of CTHRC1 with angiogenesis markers in a predictive model can be used as a functional biomarker panel to predict prognosis in LUAD.

## Supplementary information

**Additional file 1: Figure S1.** Representative immunochemistry stains for CTHRC1, VEGF and CD34. Representative image of **a** 0, **b** 0–10%, **c** 11–50%, **d** 51–75% and **e** > 75% of cancer cells stained for CTHRC1 IHC staining and **f**–**j** for five cancer cells stained categories for VEGF IHC staining in LUAD tissue samples. **k**–**o** IHC staining of different MVDs in LUAD tissue arrays. Scale bar: 50 μm. CTHRC1, collagen triple helix repeat containing 1; VEGF, vascular endothelial growth factor; MVD, microvessel density; IHC, immunochemistry; LUAD, lung adenocarcinoma. **Figure S2.** Advanced clinical stage determined the worse prognosis in LUAD. Kaplan-Meier survival curves for OS and PFS according to **a** clinical stage, **b** T, **c** N, and **d** M classification. Vertical tick marks censored subjects. Bonferroni correction was used to adjust the statistical significance level (Table S1–3). CTHRC1, collagen triple helix repeat containing 1; VEGF, vascular endothelial growth factor; MVD, microvessel density; LUAD, lung adenocarcinoma; OS, overall survival; PFS, progression-free survival. **Figure S3.** No significant difference was observed between CTHRC1-High group and CTHRC1-Low group in **a**, **b** T 3–4, **c**, **d** N 1–3 and **e, f** M1 subgroups.

**Additional file 2: Table S1.** Multiple comparison of OS and PFS between different clinical stages. **Table S2.** Multiple comparison of OS and PFS between different T classifications. **Table S3.** Multiple comparison of OS and PFS between different N classifications. **Table S4.** Multiple comparison of OS and PFS between different risk groups. **Table S5.** The ROC analyses of variables for OS and PFS.

## Data Availability

All data generated or analyzed during this current study are available from the corresponding author on reasonable request.

## Abbreviations

CTHRC1: collagen triple helix repeat containing 1
LUAD: lung adenocarcinoma
VEGF: vascular endothelial growth factor
MVD: microvessel density
OS: overall survival
PFS: progression-free survival
NSCLC: non-small cell lung cancer
FFPE: formalin-fixed and paraffin-embedded
IHC: immunohistochemistry
ROC: receiver operating characteristic
GSEA: gene set enrichment analysis
MSigDB: molecular signatures database
TCGA: The Cancer Genome Atlas
ES: enrichment score
NES: normalized enrichment score
NOM *P* value: normalized *P*-value
SD: standard deviation
AUC: areas under the curve
CI: confidence interval
PDAC: pancreatic ductal adenocarcinoma
WB: western blotting
qRT-PCR: quantitative real-time PCR
GFP: green fluorescent protein
VEGFA: vascular endothelial growth factor A
anti-VEGF: anti-vascular endothelial growth factor
HUVECs: human umbilical vein endothelial cells
ERK: extracellular-signal-regulated protein kinase
JNK: c-Jun N-terminal kinase
TEMs: Tie2-expressing monocytes
Ang-2: angiopoietin-2
HIF-1α: hypoxia-inducible factor 1α
PI3K: phosphoinositide-3-kinase
AKT: protein kinase B
mTOR: mammalian target of rapamycin
HBV: hepatitis B virus
