# Supplementary material for: High CTHRC1 expression may be closely associated with angiogenesis and indicates poor prognosis in lung adenocarcinoma patients
Source: Cancer Cell Int. 2019 Nov 29;19:318. doi: 10.1186/s12935-019-1041-5 (PMC6884781; doi:10.1186/s12935-019-1041-5)
Supplement: Supplementary file 1 — Additional file 1: Figure S1. Representative immunochemistry stains for CTHRC1, VEGF and CD34. Representative image of a 0, b 0–10%, c 11–50%, d 51–75% and e > 75% of cancer cells stained for CTHRC1 IHC staining and f–j for five cancer cells stained categories for VEGF IHC staining in LUAD tissue samples. k–o IHC staining of different MVDs in LUAD tissue arrays. Scale bar: 50 μm. CTHRC1, collagen triple helix repeat containing 1; VEGF, vascular endothelial growth factor; MVD, microvessel density; IHC, immunochemistry; LUAD, lung adenocarcinoma. Figure S2. Advanced clinical stage determined the worse prognosis in LUAD. Kaplan-Meier survival curves for OS and PFS according to a clinical stage, b T, c N, and d M classification. Vertical tick marks censored subjects. Bonferroni correction was used to adjust the statistical significance level (Table S1–3). CTHRC1, collagen triple helix repeat containing 1; VEGF, vascular endothelial growth factor; MVD, microvessel density; LUAD, lung adenocarcinoma; OS, overall survival; PFS, progression-free survival. Figure S3. No significant difference was observed between CTHRC1-High group and CTHRC1-Low group in a, b T 3–4, c, d N 1–3 and e, f M1 subgroups. [file 12935_2019_1041_MOESM1_ESM.docx]

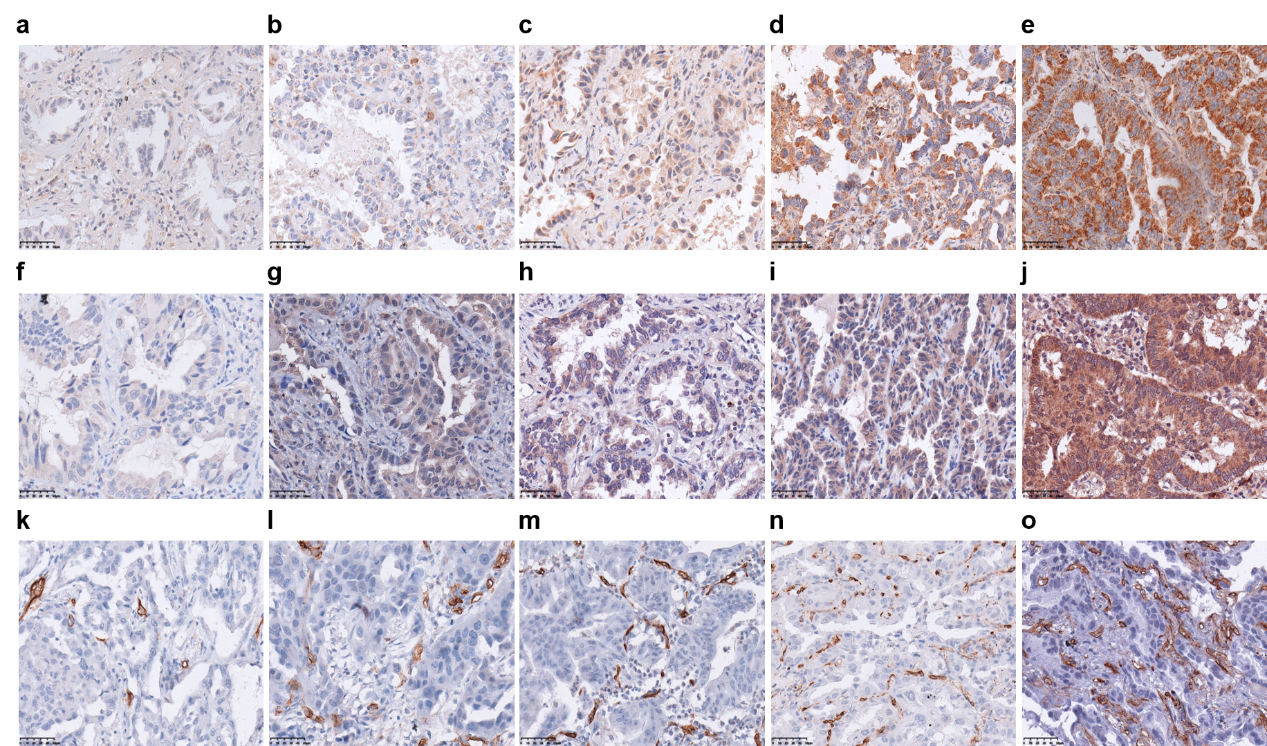
**Additional file 1**

**Figure S1.** Representative immunochemistry stains for CTHRC1, VEGF and CD34. Representative image of **a** 0, **b** 0-10%, **c** 11-50%, **d** 51-75% and **e** >75% of cancer cells stained for CTHRC1 IHC staining and **f-j** for five cancer cells stained categories for VEGF IHC staining in LUAD tissue samples. **k-o** IHC staining of different MVDs in LUAD tissue arrays. Scale bar: 50 um. CTHRC1, collagen triple helix repeat containing 1; VEGF, vascular endothelial growth factor; MVD, microvessel density; IHC, immunochemistry; LUAD, lung adenocarcinoma.


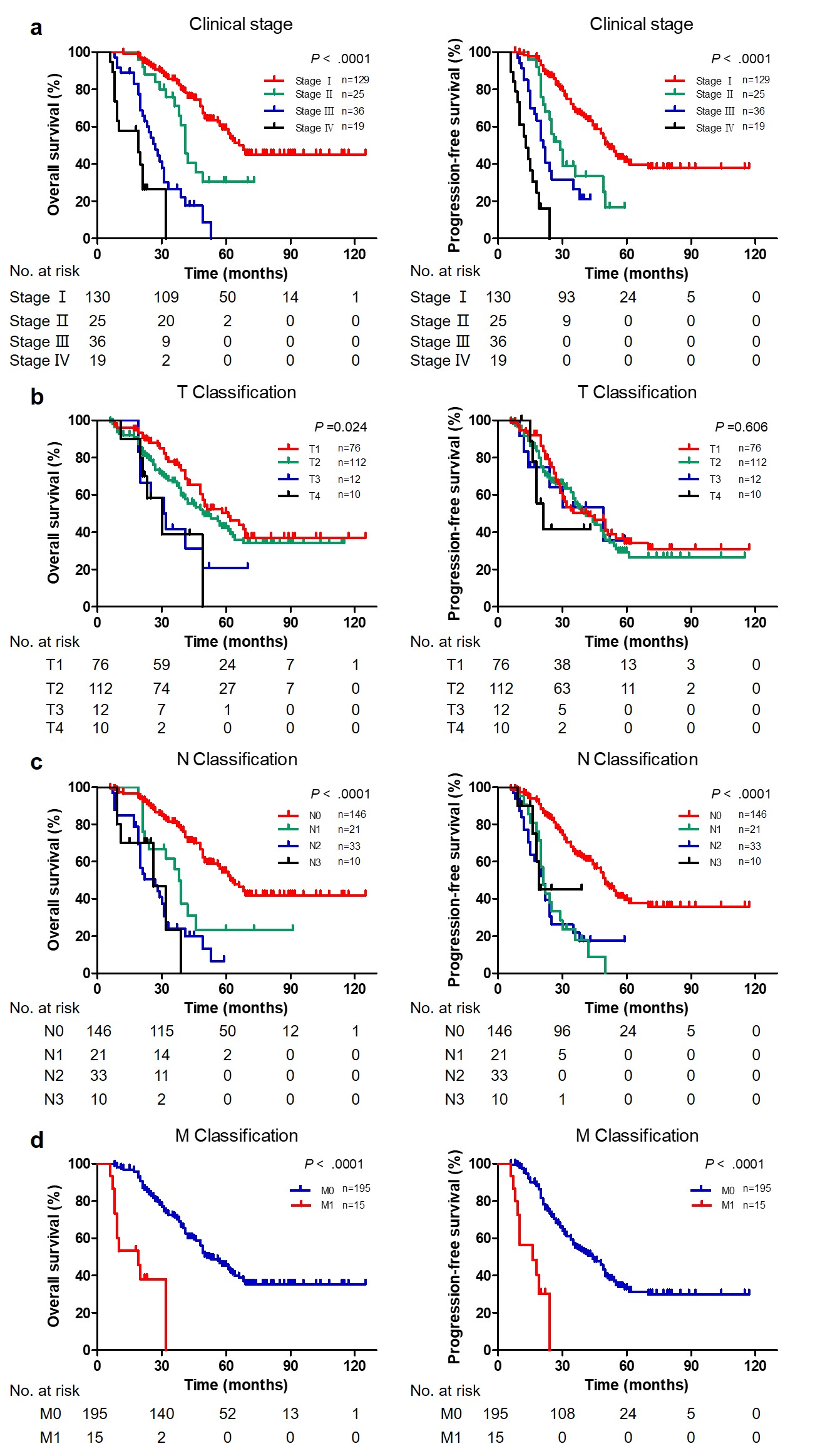


**Figure S2.** Advanced clinical stage determined the worse prognosis in LUAD. Kaplan-Meier survival curves for OS and PFS according to **a** clinical stage, **b** T, **c** N, and **d** M classification. Vertical tick marks censored subjects. Bonferroni correction was used to adjust the statistical significance level (Table S1-3). CTHRC1, collagen triple helix repeat containing 1; VEGF, vascular endothelial growth factor; MVD, microvessel density; LUAD, lung adenocarcinoma; OS, overall survival; PFS, progression-free survival.


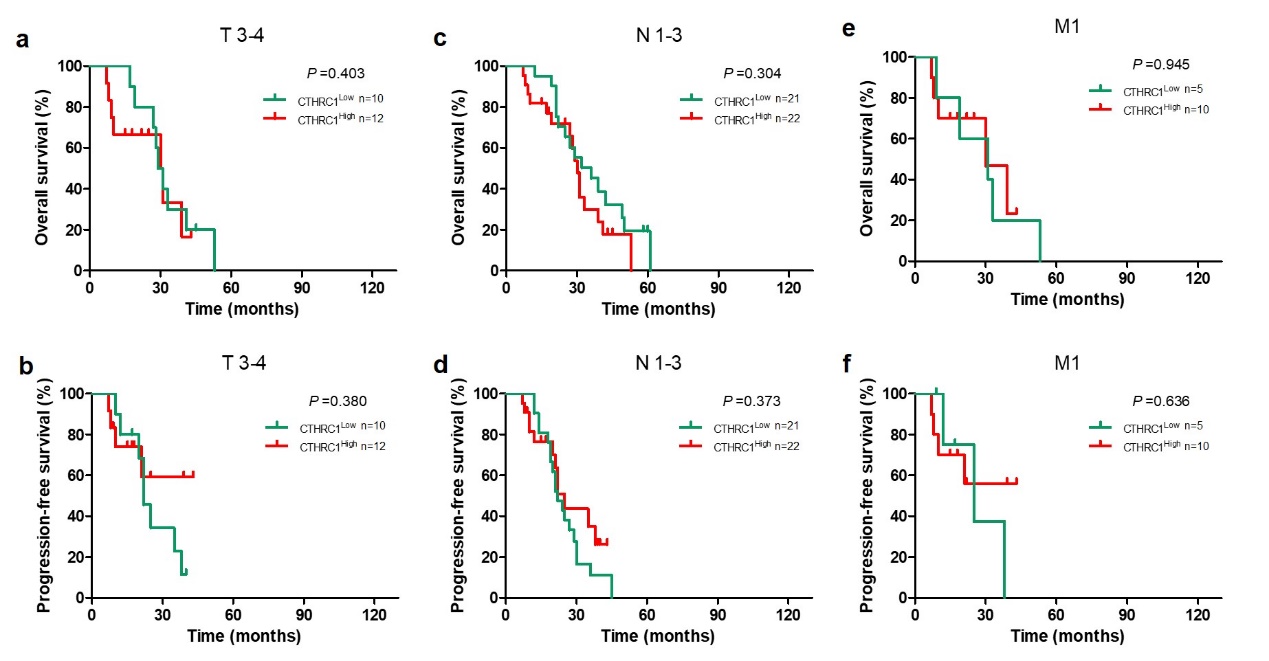


**Figure S3.** No significant difference was observed between CTHRC1-High group and CTHRC1-Low group in **a, b** T 3-4, **c, d** N 1-3 and **e, f** M1 subgroups.
