# Supplementary material for: High CTHRC1 expression may be closely associated with angiogenesis and indicates poor prognosis in lung adenocarcinoma patients
Source: Cancer Cell Int. 2019 Nov 29;19:318. doi: 10.1186/s12935-019-1041-5 (PMC6884781; doi:10.1186/s12935-019-1041-5)
Supplement: Supplementary file 2 — Additional file 2: Table S1. Multiple comparison of OS and PFS between different clinical stages. Table S2. Multiple comparison of OS and PFS between different T classifications. Table S3. Multiple comparison of OS and PFS between different N classifications. Table S4. Multiple comparison of OS and PFS between different risk groups. Table S5. The ROC analyses of variables for OS and PFS. [file 12935_2019_1041_MOESM2_ESM.docx]

**Additional file 2**

**Table S1.** Multiple comparison of OS and PFS between different clinical stages.

| **Groups** | **Overall Survival (*p*)** | **Progression-free Survival(*p*)** |
| --- | --- | --- |
| **Stage I vs. Stage II** | **0.008** | **<0.001** |
| **Stage I vs. Stage III** | **<0.001** | **<0.001** |
| **Stage I vs. Stage IV** | **<0.001** | **<0.001** |
| **Stage II vs. Stage III** | **0..001** | **0.080** |
| **Stage II vs. Stage IV** | **<0.001** | **<0.001** |
| **Stage III vs. Stage IV** | **0.007** | **0.001** |

Statistical significance (*p*<0.01) is shown in bold.

**Abbreviations:** OS, overall survival; PFS, progression-free survival.

**Table S2.** Multiple comparison of OS and PFS between different T classifications.

| **Groups** | **Overall Survival (*p*)** | **Progression-free Survival(*p*)** |
| --- | --- | --- |
| **T1 vs. T2** | 0.156 | 0.568 |
| **T1 vs. T3** | 0.205 | 0.769 |
| **T1 vs. T4** | **0.006** | 0.132 |
| **T2 vs. T3** | 0.662 | 0.948 |
| **T2 vs. T4** | 0.127 | 0.307 |
| **T3 vs. T4** | 0.400 | 0.643 |

Statistical significance (*p*<0.01) is shown in bold.

**Abbreviations:** OS, overall survival; PFS, progression-free survival.

**Table S3.** Multiple comparison of OS and PFS between different N classifications.

| **Groups** | **Overall Survival (*p*)** | **Progression-free Survival(*p*)** |
| --- | --- | --- |
| **N0 vs. N1** | **0.003** | **<0.001** |
| **N0 vs. N2** | **<0.001** | **<0.001** |
| **N0 vs. N3** | **<0.001** | 0.014 |
| **N1 vs. N2** | 0.034 | 0.913 |
| **N1 vs. N3** | 0.118 | 0.858 |
| **N2 vs. N4** | 0.932 | 0.495 |

Statistical significance (*p*<0.01) is shown in bold.

**Abbreviations:** OS, overall survival; PFS, progression-free survival.

**Table S4.** Multiple comparison of OS and PFS between different risk groups.

| **Groups** | **Overall Survival (*p*)** | **Progression-free Survival(*p*)** |
| --- | --- | --- |
| **Low risk vs. Moderate risk** | **<0.001** | **<0.001** |
| **Low risk vs. High risk** | **<0.001** | **<0.001** |
| **Moderate risk vs. High risk** | **0.001** | **<0.001** |

Statistical significance (*p*<0.017) is shown in bold.

**Abbreviations:** OS, overall survival; PFS, progression-free survival.

**Table S5.** The ROC analyses of variables for OS and PFS.

| **Variables** | **Overall Survival** | | **Progression-free Survival** | |
| --- | --- | --- | --- | --- |
|  | **AUC (95% CI)** | ***p*** | **AUC (95% CI)** | ***p*** |
| **Age** | 0.645 (0.568-0.721) | **<0.001** | 0.561 (0.482-0.640) | 0.132 |
| **Clinical stage** | 0.638 (0.563-0.713) | **0.001** | 0.596 (0.520-0.673) | **0.017** |
| **T classification** | 0.536 (0.457-0.615) | 0.370 | 0.492 (0.412-0.571) | 0.833 |
| **N classification** | 0.608 (0.531-0.684) | **0.007** | 0.568 (0.489-0.647) | 0.091 |
| **M classification** | 0.525 (0.447-0.603) | 0.537 | 0.515 (0.436-0.593) | 0.718 |
| **CTHRC1** | 0.635 (0.560-0.710) | **0.001** | 0.603 (0.527-0.680) | **0.011** |
| **VEGF** | 0.699 (0.627-0.771) | **<0.001** | 0.704 (0.632-0.776) | **<0.001** |
| **MVD** | 0.648 (0.574-0.722) | **<0.001** | 0.596 (0.519-0.673) | **0.017** |
| **The predictive model** | 0.740 (0.672-0.808) | **<0.001** | 0.691 (0.620-0.763) | **<0.001** |

Statistical significance (*p*<0.05) is shown in bold.

**Abbreviations:** OS, overall survival; PFS, progression-free survival; CTHRC1, collagen triple helix repeat containing 1; VEGF, vascular endothelial growth factor; MVD, microvessel density; ROC, receiver operative characteristic; AUC, area under the curve; CI, Confidence interval.
